# Analytical characteristics and comparative evaluation of Aptima HIV-1 Quant Dx assay with Ampliprep/COBAS TaqMan HIV-1 test v2.0

**DOI:** 10.1186/s12985-016-0627-y

**Published:** 2016-10-21

**Authors:** Angelos. Hatzakis, Helen Papachristou, Sangeetha J. Nair, Jacqueline Fortunko, Tracy Foote, HeeCheol Kim, Tashi L. Peling, Andrew J. Worlock

**Affiliations:** 1 Department of Hygiene, Epidemiology and Medical Statistics, Medical School, National and Kapodistrian University of Athens, Mikras Asias 75, GR-11527 Athens, Greece; 2 Hellenic Scientific Society for the Study of AIDS and Sexually Transmitted Diseases, Athens, Greece; 3 Hologic Inc., 10210 Genetic Center Drive, San Diego, CA 92121 USA; 4 Department of Hygiene, Epidemiology and Medical Statistics, National Retrovirus Reference Center, National and Kapodistrian University of Athens Medical School, Mikras Asias 75, GR-11527 Athens, Greece

## Abstract

**Background:**

Quantitation of HIV-RNA is critically important for diagnosis, prognosis, treatment, monitoring and assessment of infectivity in HIV-1 infection. The objective of this study was to assess performance characteristics of the Aptima HIV-1 Quant Dx assay (Aptima), a new transcription mediated amplification (TMA), fully integrated and automated assay from Hologic Inc., San Diego, CA, USA.

The analytical sensitivity, analytical specificity, precision and detection of HIV-1 subtypes were tested based on commercially available international standards or panels. A selected group of 244 anti-HIV-1 (+) plasma samples was used for comparison with Roche COBAS Ampliprep/COBAS TaqMan HIV- 1 test v2.0 (Roche CAP/CTM), (Roche Molecular Systems, Pleasanton, CA).

**Results:**

The 50 and 95 % limit of detection were estimated at 4.9 (95 % CI 3.9–5.7) and 17.6 (15.2–21.2) IU/mL respectively. The specificity was found 99.83 (99.06–99.97) %. The standard deviations and coefficient of variations for panels with 50 and 100 copies/mL (1.7 and 2 log copies/mL) were 0.14 log copies/mL (8.67 %CV) and 0.18 log copies/mL (9.91 %CV) respectively. The detection rate for Aptima and Roche assays was 220/244 (90.2 %) and 217/244 (88.9 %) respectively.

**Conclusion:**

The Aptima assay is a sensitive, specific, precise and accurate test for measuring HIV-1 viral loads and for the detection of HIV-1 infections.

## Background

Quantitation of HIV-RNA (viral load) has proven clinical utility in the diagnosis, prognosis, treatment, monitoring and the assessment of infectivity in HIV-1 infected patients [1–4].

HIV-1 is highly heterogeneous with Group M viruses responsible for the majority of HIV-1 infections worldwide. They are subdivided into nine subtypes (A-D, F-H, J, K) and numerous circulating recombinant forms (CRFs). Due to human movements and the recombination events the HIV-1 diversity is gradually increasing posing a challenge in the validity of viral load assays [5].

There are a number of commercially available viral load assays which are mainly based on real-time PCR [6–11]. These assays offer varying levels of automation based on dedicated hardware and software, including automated result interpretation as well as high throughput.

The Aptima HIV-1 Quant Dx assay (Hologic Inc. San Diego, CA, USA) is a fully integrated and automated assay for use on the Panther System. At present, the test is not approved for use in the US but is CE-IVD certified for the detection and quantitation of HIV-1. It utilizes transcription mediated amplification (TMA) involving target capture, target amplification by TMA and real-time detection of amplicons by fluorescent probes in a single tube. The Aptima HIV-1 Quant Dx assay requires only 0.5mL of sample per reaction with a dead volume as little as 0.2 mL.

The aim of this study was to assess the analytical performance characteristics of Aptima assay using commercially available panels and to evaluate it side-by-side with Roche CAP/CTM.

## Methods

### Linearity

Linearity of HIV-1 subtype B across the assay’s dynamic range was assessed using AcroMetrix HIV-1 panel from Life Technologies (Catalog number 950470 batch number 400716). This panel has 1 HIV negative panel member and 6 HIV-1 positive panel members with concentrations ranging from 1e2 to 5e6 copies/mL. The recovered concentration of each panel member was compared to the concentration value provided by the vendor. The linearity of the various subtypes of HIV-1 was confirmed by testing 5–7 dilutions of clinical specimens (HIV subtypes A1, B, C, F1 and CRF02-AG) and cultured isolates of HIV (subtypes G and A/E and group O and N). The dilutions tested for clinical specimens targeted 1e2, 5e2, 1e3, 1e4 and 1e5 copies/mL. The concentration and available volume of the clinical sample stocks did not permit testing concentrations above 1e5 copies/mL. For the clinical isolates two additional dilutions at 1e6 copies/mL and 5e6 copies/mL were also tested.

### Analytical sensitivity (limit of detection)

One vial of 2^nd^ WHO international standard for HIV-1 (NIBSC code 97/650) was reconstituted according to manufacturer’s instructions. The sample was diluted in HIV-1 negative plasma to concentrations ranging from 0 to 40 IU/mL (0, 5, 10, 15, 20, 25, 30 and 40 IU/mL). Each panel member was tested on 3 Panthers with 3 reagent lots of Aptima HIV-1 assay reagent. Ten replicates were tested for each concentration with each reagent lot on each Panther (total 90 replicates per concentration). Probit analysis was performed with the Aptima results using SAS software to calculate the 50 and 95 % detection rates.

### Analytical specificity

Specificity was assessed by testing 600 plasma specimens collected from people who were not infected with HIV-1. Testing was conducted with 2 reagent lots of Aptima assay reagents with 300 specimens being tested per reagent lot. Testing was distributed across 3 Panther instruments. All samples which gave reactive results (<1.47 log copies/mL detected or quantitative result or >7 log copies/mL) were considered as false positives in Aptima.

### Precision, reproducibility and repeatability

Within-run repeatability and between-run reproducibility of the Aptima assay was assessed by testing 8 dilutions of HIV-1 in negative plasma ranging in concentration from 50 to 10^7^ copies/mL. Each panel was tested on 3 Panthers with 3 Aptima reagent lots by three operators to a total of 27 runs. Three replicates of each panel were tested in each run. Results from all valid replicates were used for assessment of precision using SAS statistics software.

### Genetic variability and subtype detection

Subtype detection was assessed using the German NRC subtype panel composed of 21 members of which 18 belong to group M (2x A, 2x B, 2x C, 3x D, 2x CRF01_AE, 2x F, 1x G, 1x H, 1xG/H, 2x CRF02_AG), 2 to group O and 1 to group N. These panels were manufactured by National Resource Center using virus isolates originally obtained from the NIH AIDS Research and Reference Reagent Program, USA, or from the Programme EVA Centre for AIDS Reagents, NIBSC, UK. These viruses were cultivated on cells and supernatants were diluted in HIV-negative plasma to a concentration of approximately 100,000 HIV-1 RNA copies/mL. The isolates were quantified using the Roche CAP/CTM and Abbott RealTime HIV-1 (Abbott RT) assays. One replicate of each subtype panel was tested in the Aptima assay and results were compared to the Roche CAP/CTM and Abbott RT results provided by the vendor. This three-way comparison allowed for any results differing by more than 0.5 log copies/mL to be resolved.

### Assay comparison

Assessment of agreement with an established commercially available method was undertaken (Roche CAP/CTM). The Roche CAP/CTM assay targets the gag and LTR regions [12]. The assay uses at least 1 mL of specimen and reports quantifiable HIV-1 results over the range of 20 to 10,000,000 copies/mL [12]. The Aptima assay targets the pol and LTR regions. The assay uses 0.5 mL of specimen plus a minimum 0.2 ml dead volume and reports quantifiable HIV-1 results over the range of 30 to 10,000,000 copies/mL [13]. Both Aptima and Roche assays report specificity of 100 % in their respective package inserts [12, 13]. Two hundred forty-four patient plasma anti-HIV-1 (+) samples were collected, aliquoted and frozen. Samples were selected based on an historical HIV viral load result based on Roche AmpliPrep/COBAS Taqman HIV-1 to provide results across the range of both Roche and Aptima assays. One replicate of each sample was tested in Aptima and Roche assays following the manufacturer’s instructions. One hundred seventy-four samples which had quantitative results in both assays were used for linear regression and Bland-Altman analysis. These included different HIV-1 subtypes and recombinants.

### Statistical methods

The 50 and 95 % positivity rates were estimated using Probit analysis using the normal model and analysis software from the SAS Institute Cary, NC, USA.

The method comparison simple linear regression and Bland Altman analysis were performed using Analyse-it Software, Leeds, United Kingdom.

Comparison of the Aptima HIV-1 results to Roche CAP/CTM was performed using McNamara’s test with analysis software from the SAS Institute Cary, NC, USA.

Linearity was measured using the R^2^ of the Pearson correlation coefficient calculated using Microsoft Excel (WA, USA).

## Results

### Linearity

**Fig. 1:**
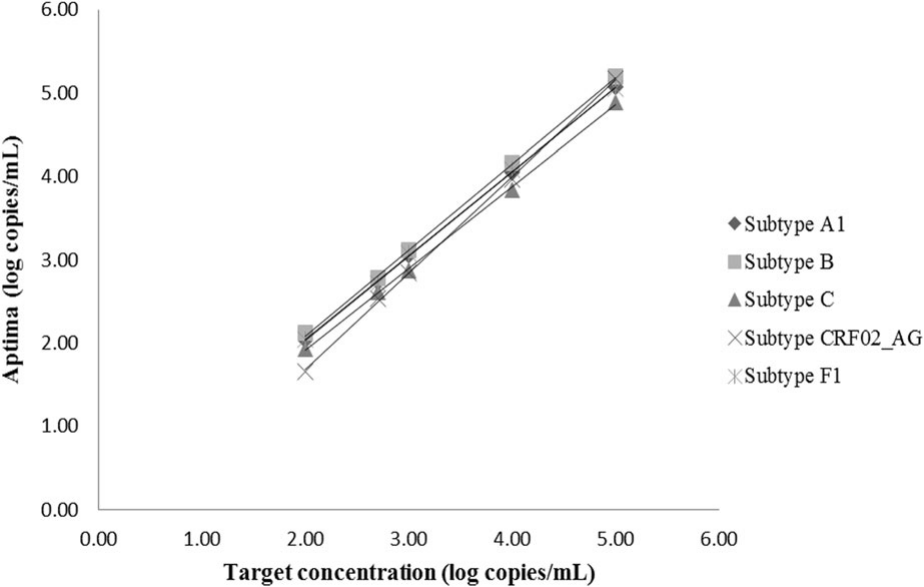
Linearity of Aptima based on clinical specimens representing different HIV subtypes

**Fig. 2:**
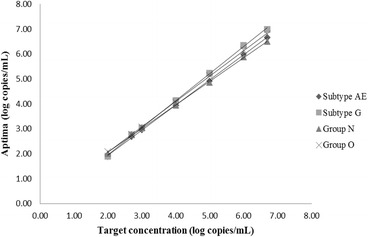
Linearity of Aptima based on﻿ cultured isolates representing different groups or subtypes of HIV

As seen from Table 1 all members of the HIV-1 subtype B AcroMetrix linearity panel, ranging in concentration from 1e2 to 5e6 copies/mL, recovered within 0.25 logs of the target concentration. The results in Aptima were also linear for all the major groups, subtypes and recombinants of HIV (Figs. 1 and 2). The R^2^ values of the plots in Figs. 1 and 2 were all above 0.999 as shown in Table 2.

**Table 1:** Recovery of AcroMetrix subtype B Linearity Panel in Aptima HIV Quant Dx

Target concentration (log copies/mL)	Average Aptima (log copies/mL)	Average Aptima-Target
Not detected	Not detected	Not detected
2.00	2.05	0.05
2.70	2.60	−0.10
3.70	3.65	−0.05
4.70	4.86	0.16
5.70	5.93	0.23
6.70	6.92	0.22

**Table 2:** R^2^ Values Linearity of Plots Aptima Based on Clinical Specimens Representing Different HIV Groups or Subtypes

HIV Group or Subtype	R^2^
A	1.0000
B	0.9996
C	0.9995
AG	0.9996
F1	0.9995
AE	0.9996
G	0.9994
N	0.9994
O	0.9997

### Analytical sensitivity of Aptima

By use of HIV-1 2^nd^ International WHO standards diluted in plasma, the 50 and 95 % limit of detection and 95 % confidence intervals (shown in brackets) were estimated by Probit analysis as 4.9 (3.9–5.7) and 17.6 (15.2–21.2) IU/mL, respectively (Table 3).

**Table 3:** Assessment of analytical sensitivity of Aptima HIV-1 Quant by dilutions of HIV-1 2nd international WHO standard diluted in plasma

Panels IU/mL	Number	Number positive	% Positive
0	89	0	0 %
5	89	46	52 %
10	90	73	81 %
15	90	84	93 %
20	90	88	98 %
25	89	87	98 %
30	90	89	99 %
40	89	89	100 %

### Analytical specificity

The combined analytical specificity using 600 HIV-1 negative specimens from two different lots was estimated 99.83 (95 % CI of 99.06–99.97 %) (Table 4).

**Table 4:** Assessment of analytical specificity of Aptima HIV-1 Quant Dx assay by two lots of HIV-1 negative samples

Reagent lot	N	Negative	Specificity	LCI	UCI
lot 1	300	299	99.67	98.14	99.94
lot 2	300	300	100	98.74	100.00
Combined	600	599	99.83	99.06	99.97

### Precision, reproducibility and repeatability

The CVs for inter-instrument, inter-operator, inter-lot, inter-day, inter-run and intra-run variation were 0–4.52 %, 0–1.99 %, 0–0.77 %, 0–0.35 %, 0–5.6 % and 0.44–8.14 %, respectively. The total CVs for panels of 50 copies/mL, 10^2^, 10^3^, 10^4^,10^5^, 10^6^, 10^7^ copies/mL were estimated 8.67 %, 9.91 %, 4.08 %, 1.77 %, 1.35 %, 0.92 %, 0.9 % respectively (Table 5).

**Table 5:** Assessment of inter-instrument, inter-operator, inter-lot, inter-day, inter-run, intra-run and total variability of Aptima HIV-1 Quant Dx in various HIV-1 RNA concentrations

			Inter-instrument		Inter-operator		Inter-lot		Inter-day		Inter-run		Intra-run		Total	
Panel	N	Mean	SD	CV (%)	SD	CV (%)	SD	CV (%)	SD	CV (%)	SD	CV (%)	SD	CV (%)	SD	CV (%)
		Log														
5E1 copies/mL	41^a^	1.66	0.075	4.52	0.033	1.99	0	0	0	0	0	0	0.119	7.12	0.144	8.67
1E2 copies/mL	74^a^	1.82	0	0	0	0	0.014	0.77	0	0	0.102	5.6	0.148	8.14	0.18	9.91
1E3 copies/mL	81	2.75	0.035	1.26	0	0	0	0	0	0	0.041	1.5	0.098	3.57	0.112	4.08
1E4 copies/mL	81	3.81	0.011	0.28	0.013	0.35	0	0	0.013	0.35	0.05	1.31	0.039	1.04	0.067	1.77
1E5 copies/mL	81	4.96	0.01	0.21	0	0	0	0	0	0	0.042	0.84	0.051	1.03	0.067	1.35
1E6 copies/mL	78^b^	6	0.008	0.14	0.001	0.02	0	0	0	0	0.048	0.79	0.027	0.44	0.055	0.92
1E7 copies/mL	81	6.89	0	0	0	0	0.026	0.38	0	0	0.033	0.47	0.046	0.67	0.062	0.9

Note: 1. Variability from some factors may be numerically negative, which can occur if the variability due to those factors is very small. When this occurs, SD = 0 and CV = 0 %

^a^the 50 and 100 copies/mL sample has lower N because some results were below the limit of quantification

^b^3 results were invalid and not included in the analysis

### Genetic variability and subtype detection using HIV-1 viral isolates

Twenty one viral isolates were used for comparison of Aptima Roche CAP/CTM and Abbott RT. Aptima consistently quantified slightly higher compared to target values based on Roche CAP/CTM with differences ranging from 0.01 to 0.79 log copies/mL (Table 6). The differences between Aptima and Abbott RT results ranged from −0.12 to 1.04 log copies/mL.

**Table 6:** Subtype detection by aptima HIV-1 quant and roche CAP/CTM in viral isolates diluted to 10^5^ copies/mL of HIV-RNA

Isolate	Subtype/Group	Aptima log Copies/mL	Roche CAP/CTM log Copies/mL	Aptima – Roche CAP/CTM	Abbott RT (Log Copies/mL)	Aptima- Abbott RT
92UG029	A	5.92	5.57	0.35	5.60	0.32
00KE_KER2018	A	5.11	4.98	0.13	5.02	0.09
92TH026	B	5.54	5.49	0.06	5.66	-0.12
90TH_BK132	B	5.57	5.37	0.21	5.36	0.21
92BR025	C	5.51	5.26	0.25	5.32	0.18
99ET_14	C	6.38	6.12	0.26	5.91	0.48
92UG021	D	5.47	5.45	0.03	5.51	-0.03
92UG035	D	6.02	5.61	0.40	5.77	0.25
92UG024	D	5.74	5.43	0.30	5.61	0.13
92TH022	CRF01_AE	5.25	not available	not available	5.26	-0.01
92TH009	CRF01_AE	5.33	5.08	0.25	5.25	0.08
93BR029	F	5.15	5.16	0.01	5.18	-0.03
93BR020	F	5.59	not available	not available	5.19	0.40
RU570	G	5.77	not available	not available	5.70	0.08
VI557	H	5.95	not available	not available	5.43	0.51
01CM.0005BBY	CRF02_AG	4.89	4.77	0.12	4.68	0.21
01CM.0008BBY	CRF02_AG	5.23	5.00	0.23	4.95	0.27
VI525	G/H	5.21	4.42	0.79	4.94	0.27
YBF 30	N	6.20	5.72	0.48	5.17	1.04
MVP5180	O	4.32	not available	not available	4.23	0.09
CA-9	O	4.76	4.75	0.01	4.32	0.43

### Comparison of Aptima with Roche CAP/CTM using clinical samples

Aptima quantified 220/244 (90.1 %) and Roche 217/244 (88.9 %) of the clinical samples. Eleven samples were Roche (+), Aptima (−) and 14 samples Aptima (+), Roche (−) (Table 7). One hundred seventy four samples were quantified by both methods (McNemar’s *p* = 0.5485).

**Table 7:** Qualitative 2 × 2 comparison of Aptima and Roche CAP/CTM

	Aptima			
		**+**	**-**	Total
Roche CAP/CTM	**+**	206	11	217
	**-**	14	13	27
	Total	220	24	244

The *p*-value for McNemar’s test is 0.5485 indicating that the difference in positivity between Aptima and Roche for the type of samples tested is not statistically significant

**Fig. 3:**
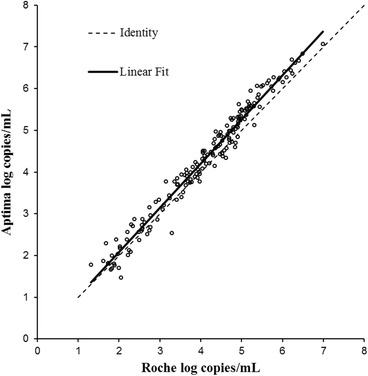
Correlation between Aptima and Roche CAP/CTM in 174 samples quantified by both assays

**Fig. 4:**
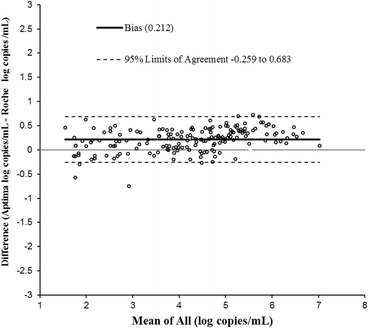
Bland Altman plot to assess the agreement between Aptima HIV-1 Quant Dx and Roche CAP/CTM in 174samples quantified by both assays

Linear regression analysis showed excellent correlation between Aptima and Roche (*R* ^2^ = 0.971). The slope was 1.06 (Fig. 3).which was statistically significant (*p*-value < 0.0001). The intercept was −0.034 which was not statistically significant (*p*-value 0.566). By Bland-Altman analysis (Fig. 4), the bias was 0.212 log copies/mL with 95 % agreement intervals of −0.259 to 0.683.

Forty seven of 174 quantifiable by both assays were subtype B, 99 subtype A, 11 Subtype C, 3 subtype G and 14 were CRFs.

**Fig. 5:**
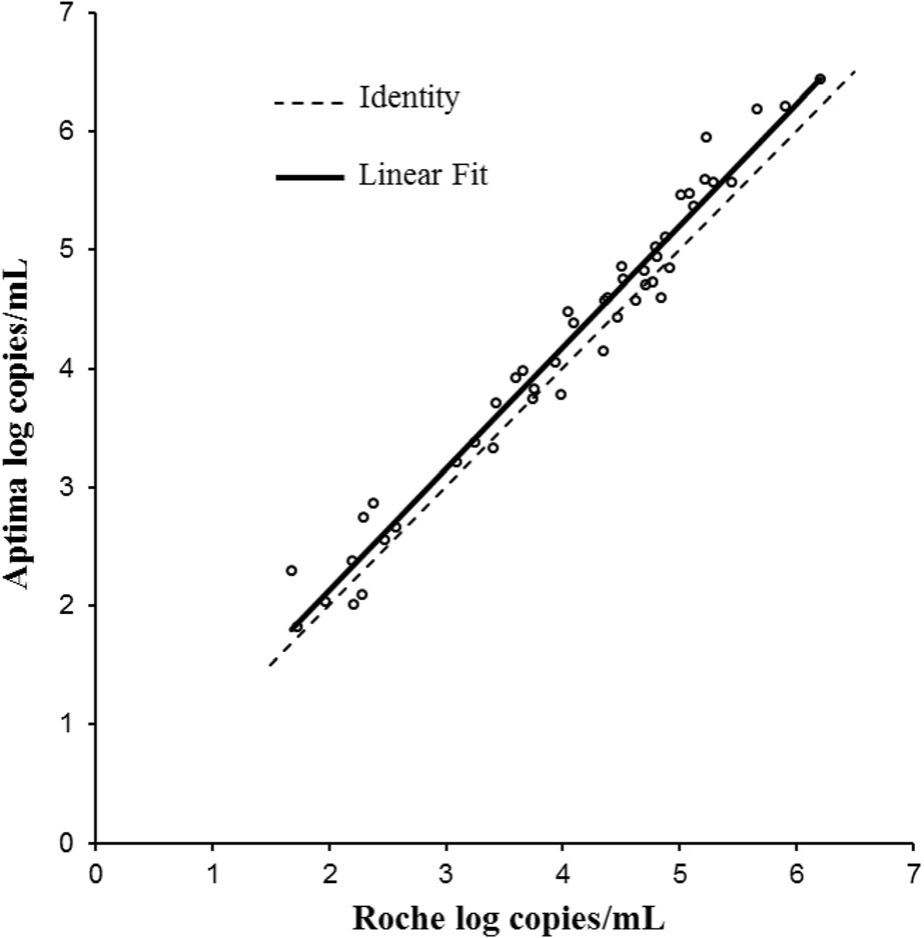
Correlation between Aptima and Roche CAP/CTM for 47 samples quantified as subtype B by both assays

**Fig. 6:**
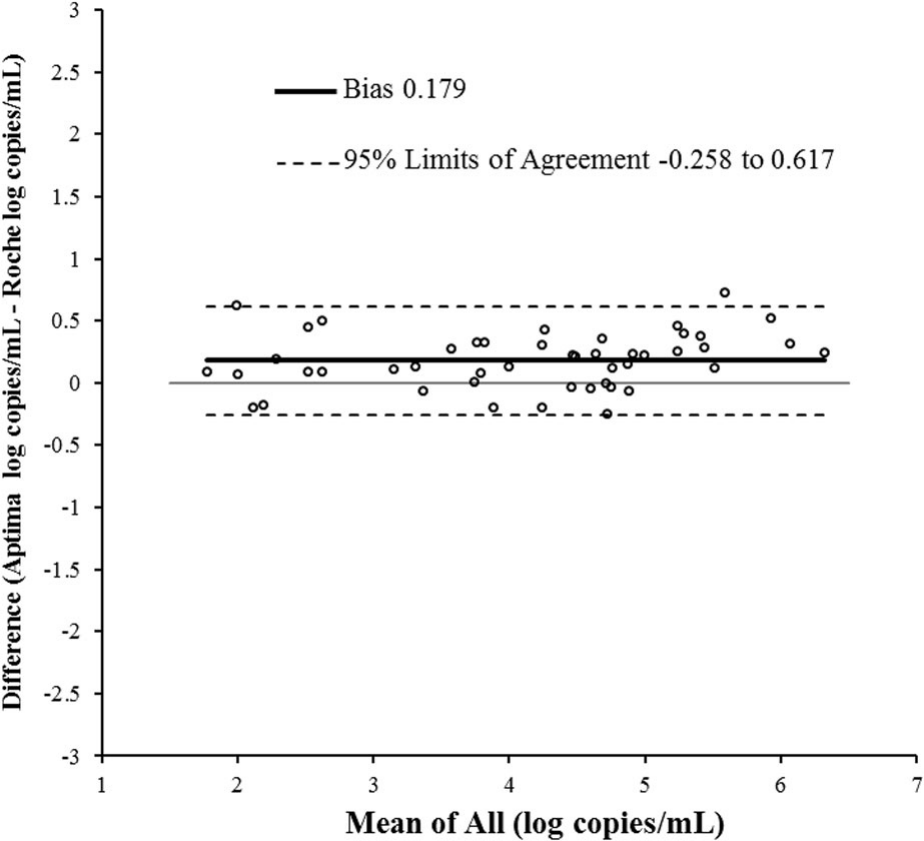
Bland Altman plot to assess the agreement between Aptima and Roche CAP/CTM in 47 subtype B samples quantified by both assays

Linear regression for subtype B is shown in Fig. 5. The R^2^ was 0.968. The slope was 1.02 (Fig. 5) which was statistically not significant (*p*-value 0.384). The intercept was 0.08 which was not statistically significant (*p*-value 0.494). By Bland-Altman analysis (Fig. 6) the bias was 0.179 log copies/mL with 95 % agreement intervals of −0.258 to 0.617.

**Fig. 7:**
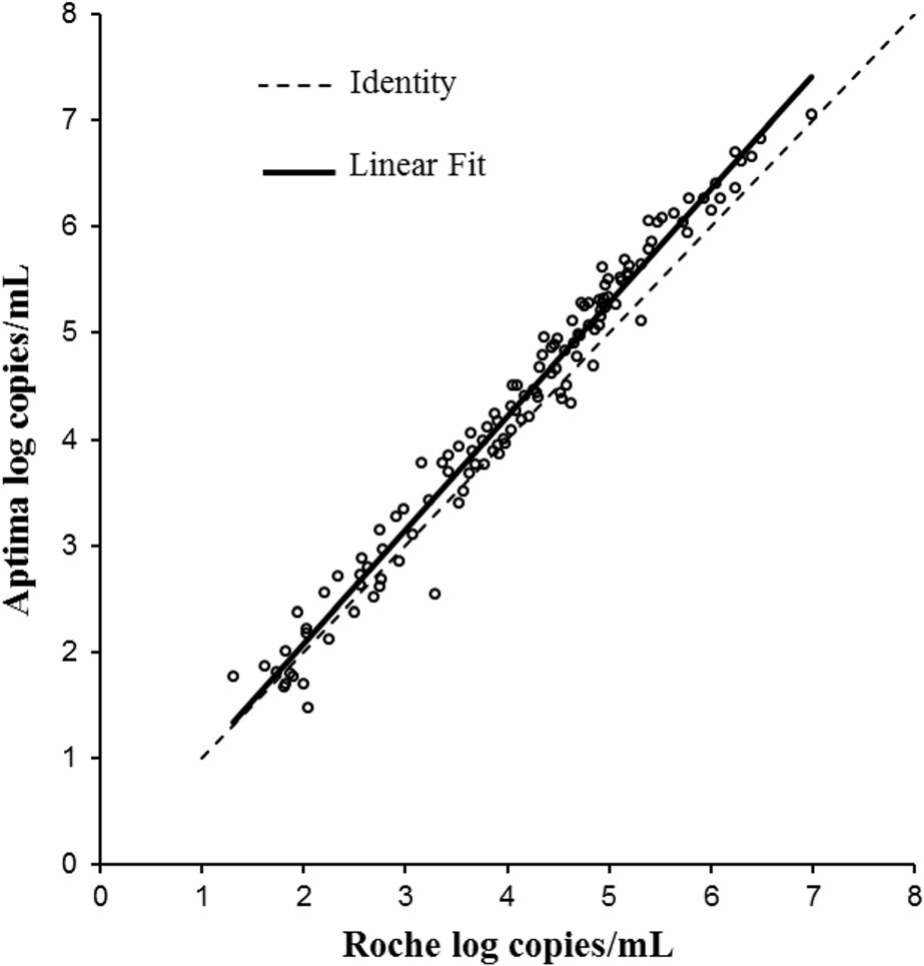
Correlation between Aptima and Roche CAP/CTM in 127 samples of non-B subtype quantified by both assays

**Fig. 8:**
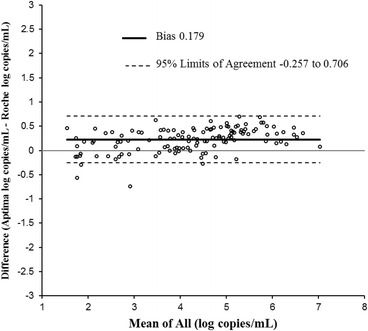
Bland Altman plot to assess the agreement between Aptima HIV-1 and Roche CAP/CTM in 127 samples of non-B subtypes quantified by both assays

Linear regression for non-B subtypes is shown in Fig. 7. The R^2^ was 0.972. The slope was 1.07 (Fig. 7) which was statistically significant (*p*-value <0.0001). The intercept was −0.07 which was not statistically significant (*p*-value 0.327). By Bland-Altman analysis (Fig. 8) the bias was 0.179 log copies/mL with 95 % agreement intervals of −0.257 to 0.706.

## Discussion

Detection and quantification of HIV-1 is important not only for diagnosis of HIV-1 infections but also for management of HIV-1 patients [14]. High viral loads are correlated with an increased risk of clinical progression of HIV-associated disease, and reductions in plasma virus levels may be associated with decreased risk of clinical progression [15–17]. To ensure the best management of HIV positive patients it is important that the test used to measure HIV-1 viral loads is sensitive, specific, accurate and precise. As shown in this study the Aptima assay on the Panther System is a highly sensitive, specific and reproducible test for the detection and quantitation of HIV-RNA across a wide dynamic range.

As evident from the results of the AcroMetrix linearity panel (Table 1) and dilutions of HIV subtype clinical samples and isolates (Figs. 1 and 2) good linearity was demonstrated across the assay range for all subtypes of HIV in Aptima. The R ^2^ values for all the HIV groups and subtypes tested were 0.999 or greater as shown in Table 2. The HIV subtypes included A, B, C, AG, F1, AE and G together with groups N and O. This shows that the Aptima assay remains linear across a genetically diverse sample population. This confirms the results shown in the Aptima package insert [13] for HIV-1 groups M, N and O.

The data presented in the package inserts for Roche CAP/CTM [12] and Aptima [13] show the assays are standardized to two different WHO standards (the 2^nd^ and 3^rd^ lot of the WHO standard respectively). To enable a direct comparison of analytical sensitivity between the two assays, the data on analytical sensitivity presented in this paper was based on dilutions of the 2^nd^ WHO international standard run in the Aptima assay. The data is presented in IU/mL for this analysis to avoid any issues converting between IU/mL to copies/mL for assays standardized using two different WHO standards. The 95 % limit of detection (LoD) of Aptima was found to be 17.6 (95 % CI 15.2–21.2) IU/mL. This is slightly more sensitive than the reported value for Roche CAP/CTM assay at 33 IU/mL [12, 18]. The higher sensitivity of Aptima compared to Roche CAP/CTM has been reported by other investigators [19]. Table 7 shows 14 Aptima positive, Roche CAP/CTM negative results compared to 11 Aptima negative, Roche CAP/CTM positive results. Although this was not statistically significant it suggests the Aptima assay is more sensitive than the Roche CAP/CTM. The high sensitivity of the Aptima assay indicates that it will be useful for both as an aid in clinical management of and diagnosing patients with HIV-1 infections.

It is also important for effective patient management that the specificity of the assay is high. In this study specificity was 99.83 % when testing 600 plasma samples. A single sample from 600 gave a positive result. The positive result was reported as “<30 copies/mL detected” and so was below the lower limit of quantitation for the assay. Since the result was not quantifiable it represents a very low level of HIV-1 between 1 and 30 copies/mL.

The subtype detection was evaluated using the NRC subtype panel composed of 21 panel members targeted at approximately 100,000 HIV-1 RNA copies/mL. The isolates included subtype A, B, C, D, F, G and H as well as circulating recombinants. Viral load results for the Aptima assay were slightly higher than those for Roche CAP/CTM but were within 0.5 log copies/mL in all cases with one exception. This isolate which was a recombinant of G and H had a viral load result from Aptima that was 0.79 log copies/mL higher than Roche CAP/CTM. Abbott RT results were obtained for this panel member which allow a comparison to be made to a third assay. The Aptima result was within 0.27 logs of the Abbott RT result. This suggests that the Roche CAP/CTM result is low. Results for the remaining panel members were available from the vendor for the Abbott RT assay. The Aptima results were within 0.5 logs of the Abbott RT result for 19 of the 21 samples. For 2 samples the Abbott RT results were 0.51 (subtype H) and 1.04 (Group N) logs lower than Aptima. The Aptima result was within 0.5 logs of the Roche CAP/CTM result for the Group N sample suggesting that the Abbott RT result is low. Roche CAP/CTM results were unavailable for the subtype H sample. This data confirms the results in the Aptima package insert [13] showing equal HIV-1 group and subtype quantitation.

In the head to head comparison of Aptima and Roche CAP/CTM high correlation was seen between the results in the two assays. The slope was 1.06 for all quantifiable results. The Aptima assay gave slightly higher results compared to Roche CAP/CTM at the higher range of the assay and this contributed to the slope. These differences are not clinically significant and are similar to other studies [20, 21]. The average bias between Aptima and Roche CAP/CTM was 0.212 log copies/mL which was mainly driven by higher Aptima values at the higher range of the assay. This small bias is not clinically significant. HIV subtype B is the most prevalent subtype in Western Europe [22]. The clinical specimen data set was broken into two subsets for further analysis (subtype B samples and non subtype B samples). Compared to Roche CAP/CTM, the Aptima assay had a slope of 1.02 for subtype B and 1.07 for non-subtype B samples. The Bland Altman plot showed a slight positive bias for this data of 0.179 log copies/mL for both sets of data but this is not clinically significant. The higher slope seen for non subtype B compared to subtype B suggests that the high proportion of non B subtypes in the overall comparison to Roche CAP/CTM contributed to the higher slope. In general the results in this study are favorably compared with previous comparisons with the Aptima assay and assays such as Abbott RealTi*m*e, Roche CAP/CTM, COBAS® TaqMan® HIV-1 Test v2.0 for use with the High Pure System (HPS/CTM), Artus HIV-1 on the Qiagen Rotor-Gene Q and Nuclisens EasyQ v2 (BioMerieux SA, Marcy L’Etoile, France) [13, 23–25].

There are significant advantages when running the Aptima assay for lab work flow. The Panther system is fully automated so that samples can be placed directly on the system without prior nucleic acid extraction. This system allows random access testing of various analytes, processing up to 320 samples in an 8-hour shift and returning of results in about 2.5 hour﻿s. This enables high flexibility to adapt to low or high throughput testing. In addition, as reported by Sam et al. [21] the cumulative hands-on time required for specimen processing and maintenance steps were consistently shorter with the Panther than with the Abbott m2000 system.

## Conclusion

In conclusion, the Aptima assay has excellent comparative performance with Roche CAP/CTM. The assay is a good choice for clinicians in Europe for the detection and monitoring of patients infected with HIV-1 as it has CE–IVD certification. At present, the test is not approved for use in the US. The high sensitivity along with close agreement to Roche CAP/CTM suggests re-baseline of patients is not required when switching to the Aptima assay for HIV-1 testing.

## Abbreviations

Abbott RT: Abbott RealTi*m*e
AIDS: Acquired immune deficiency syndrome
Aptima Assay: Aptima HIV-1 Quant Dx assay
CRF: Circulating recombinant forms
CV: Coefficient of variation
HIV: Human immunodeficiency virus
LoD: Limit of detection
mL: Millilitre
NIBSC: National Institute for Biological Standards and Control
NRC: National Reference Centre
PCR: Polymerase chain reaction
RNA: Ribonucleic acid
Roche CAP/CTM: COBAS Ampliprep/COBAS TaqMan HIV-1 test v2.0
TMA: Transcription mediate amplification
WHO: World Health Organization
